# Divergent thinking as a predictor of life skills in patients with schizophrenia: Evidence from the modified Tinkertoy Test

**DOI:** 10.1002/pcn5.222

**Published:** 2024-07-01

**Authors:** Yasuhisa Nakamura, Reiko Miyamoto, Akihiro Koreki, Sachiko Anamizu, Masaru Mimura

**Affiliations:** ^1^ Department of Rehabilitation, Division of Occupational Therapy, Faculty of Health Sciences Nihon Fukushi University Handa City Aichi Prefecture Japan; ^2^ Division of Occupational Therapy, Faculty of Health Sciences Tokyo Metropolitan University Tokyo Japan; ^3^ Department of Psychiatry NHO Shimofusa Psychiatric Medical Center Chiba Japan; ^4^ Department of Psychiatry NHO Tochigi Medical Center Tochigi Japan; ^5^ Department of Human and Social Sciences Tokyo Metropolitan University Tokyo Japan; ^6^ Department of Neuropsychiatry Keio University School of Medicine Tokyo Japan

**Keywords:** cognition, neuropsychological tests, patients, regression analysis, schizophrenia

## Abstract

**Aim:**

Patients with schizophrenia often exhibit poor life skills, posing significant clinical challenges. Life skills comprise cognitive functions crucial for planning daily activities, including divergent thinking. However, the cognitive deficits contributing to these diminished skills among patients with schizophrenia are underexplored. This study introduces a modified Tinkertoy Test (m‐TTT) to investigate the correlation between life skills, divergent thinking, and psychological assessment tools in patients with schizophrenia.

**Methods:**

Fifty‐two patients with schizophrenia, alongside a control group, matched for sex, age, and education, were evaluated using psychological assessment tools. For the patient group, the Life Skills Profile (LSP) and Positive and Negative Syndrome Scale were administered to measure functional abilities and psychiatric symptoms, respectively. Additionally, duration of disease and antipsychotic daily dosage levels were assessed exclusively in the patient group. Both groups were evaluated with the m‐TTT, Idea Fluency Test (IFT), Design Fluency Test (DFT), and Brief Assessment of Cognition in Schizophrenia (BACS) to comprehensively assess cognitive functions. A stepwise multiple regression model was conducted to identify significant correlates of LSP total score among the patient group.

**Results:**

The schizophrenia group scored notably lower than the neurotypical controls on the m‐TTT, IFT, DFT, and BACS. Our stepwise multiple regression analysis highlighted that the LSP total score was significantly correlated with the total m‐TTT score and presence of negative symptoms.

**Conclusion:**

Divergent thinking could be a crucial factor in the life skills of individuals with schizophrenia. Rehabilitation programs based on this cognitive function might enhance their daily living capabilities.

## INTRODUCTION

Schizophrenia often leads to challenges in daily activities, including personal hygiene, household chores, and social interactions.^1^, ^2^, ^3^ Such challenges in life skills among patients with schizophrenia can be attributed to cognitive dysfunction and psychiatric symptoms.^4^, ^5^ Divergent thinking (DT), a key cognitive function, plays a crucial role in problem‐solving and adaptation to community living.^6^

Previous studies have highlighted the nuanced relationship between community adaptation and different types of thinking in schizophrenia. DT, defined as the generation of a wide array of ideas from a singular starting point,^7^ emerges as a significant factor in community‐living adaptation. Concurrently, convergent thinking, which is about building logical connections and methodically reaching an optimal conclusion, is also viewed as important in this regard.^8^ However, a holistic consensus regarding this topic remains elusive. Recent reviews have indicated that DT is strongly influenced by inhibitory controls and the ability to toggle between inhibitory and noninhibitory responses based on the task's demand.^9^

Historically, the evaluation of DT in schizophrenia has employed the Idea Fluency Test (IFT) for verbal assessments, and both the Design Fluency Test (DFT) and Torrance Test of Creative Thinking for nonverbal evaluations.^10^ In this study, we pivot toward the Tinkertoy Test (TTT) as a fresh perspective to assess DT.^11^ Originally conceived by Lezak^12^ for executive function evaluation, the TTT challenges participants to craft a structure using a set of 50 Tinkertoy pieces. Given its open‐ended nature, the TTT demands that participants autonomously plan, initiate, and execute a potentially complex task.^12^ Past studies suggest that TTT results might indicate foundational life skills, such as spontaneous initiation and monitoring of daily activities.^13^ Comparisons have been drawn between the TTT scores of neurotypical individuals and those associated with conditions such as brain injury,^14^, ^15^, ^16^ Alzheimer's disease,^17^, ^18^ poststroke syndrome,^19^ and schizophrenia.^20^ Furthermore, the potential of the TTT scores to forecast employability and aptitude to engage in social tasks was explored.^21^ However, traditional TTT scoring, which focuses on a test's end product, does not capture the full extent of DT. Recognizing this gap, we introduced a modified and refined version of the TTT (m‐TTT) that includes elements of task initiation, planning, execution, and score monitoring. Subsequently, we gauged the reliability and validity of the m‐TTT as a test for DT in patients with schizophrenia.^20^

Our previous research indicated that patients with schizophrenia scored lower on the m‐TTT compared to their neurotypical counterparts.^20^ In this study, we thoroughly investigated the relationship between DT and life skills in patients with schizophrenia. By clarifying the predictive value of DT on life skills, this research can pinpoint potential areas of intervention for individuals with schizophrenia.

## METHODS

### Participants

Fifty‐two Japanese outpatients with schizophrenia (36 men and 16 women) and 51 neurotypical control participants (27 men and 24 women) were recruited for this study (Table 1). Patients with schizophrenia were diagnosed by psychiatrists according to the diagnostic criteria in the *Diagnostic and Statistical Manual of Mental Disorders*, Fifth Edition.^22^ The mean age of the patients was 40.9 years, and the mean number of years of education was 14.2. The mean duration of illness was 16.1 years. The patients with schizophrenia who participated in the study were receiving pharmacological treatment as outpatients, and their mental functions were stable, allowing them to live in the community.

**Table 1:** Demographic and clinical characteristics of the Japanese patients with schizophrenia and control groups.

		Schizophrenia group	Neurotypical control group
		(*n* ＝ 52)	(*n* ＝ 51)
Age (years)		40.9 (9.5)	40.0 (10.7)
Sex	Male/female	36/16	27/24
Education	Years	14.2 (1.4)	14.5 (1.1)
JART	IQ	100 (12.8)	104 (8.8)
Duration of disease	Years	16.1 (8.3)	
Antipsychotics daily dosage level (mg/day)	Chlorpromazine equivalent	540 (251.0)	

Values are numbers or means (standard deviations in parentheses).

Abbreviation: JART, Japanese Adult Reading Test.

The researchers obtained information on participants' age, gender, educational history, and antipsychotic daily dosage levels from the hospital medical records. No significant differences in sex, age, or educational level were observed between the two groups.

All participants were right‐handed according to the Edinburgh Inventory.^23^ None of them had a history of alcoholism, drug abuse, or serious neurological illness. None of the neurotypical control participants reported any previous psychiatric disorders or familial history of psychosis.

### Measures

#### Assessment of DT

Three tests were administered to assess the DT: m‐TTT, IFT, and DFT. The m‐TTT was designed to measure the quality of DT without constraining the participants to reproduce models or follow certain instructions. In the fluency tests, the examiners prompted each participant to produce as many responses as possible within a specified time for both verbal and nonverbal design tasks.

##### Modified Tinkertoy Test

**Figure 1:**
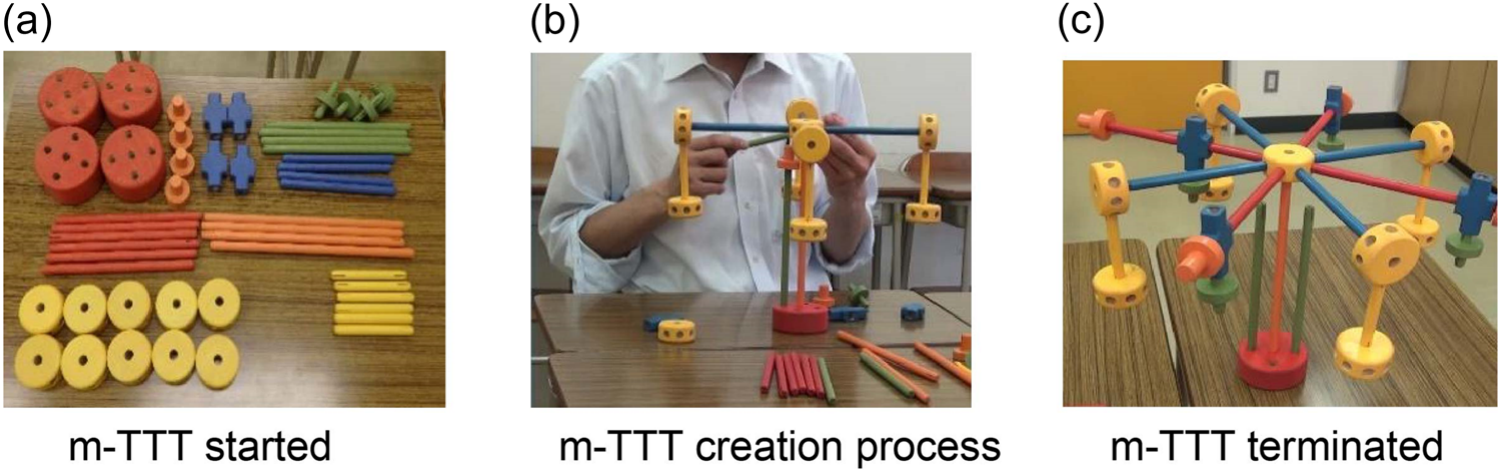
Modified Tinkertoy Test (m‐TTT) procedure.

This study used an m‐TTT.^11^, ^12^ A Tinkertoy is a toy set of construction materials for children. Originally, the TTT was conceptualized as an executive functioning assessment. Nonetheless, we shifted our focus to the free‐composition aspect of the TTT and assessed its potential as a DT test. To this end, we incorporated a creation process score into the traditional scoring method, leading to a modified version of the TTT (the m‐TTT), during which participants were asked to create a structure using 50 pieces from a Tinkertoy set consisting of various wooden pieces that could be freely assembled. The sole instruction provided to them was to “make whatever they wanted.” Participants were required to spend more than 5 min to complete the work, and upon completion, they were to declare it finished on their own. If a participant declared completion in less than 5 min, they were encouraged to think a little more. The specific details of the test procedures are illustrated in Figure 1. The test was unstructured, allowing participants to independently initiate, plan, and execute potentially complex activities. The criterion‐related validity of the cumulative score from this m‐TTT has been corroborated with both verbal and nonverbal DT scores. The creation process score demonstrated internal consistency when paired with conventional evaluation methods. This means that the m‐TTT is not simply a combination of two independent evaluation criteria (the complex score and the creation process score). Rather, they work together as facets of divergent thinking. Correlating them leads to an enhanced accuracy in DT measurement.^20^

Upon completion, the examiner inquired what the constructed object represented. If the construction did represent something—usually an object with a specific name—it was evaluated for how appropriately it aligned with the given name or concept. Subsequent questions asked in this study included, “How did you plan your approach after examining the parts?,” “How did you adapt when you were unable to create the object you had planned?,” and “What does each part of the construction represent?” Based on the responses to these questions and additional observations, the examiner graded the process of creation.

In the original scoring system, as detailed in Table 2, points were allocated based on the following criteria: (1) made constructions (MC); (2) the total number of pieces used (NP); (3) whether the construction was appropriately named based on its appearance (Name); (4a) functional mobility, such as working wheels (Mob); (4b) moving parts (MOV); (5) three‐dimensional complexity (3D); (6) freestanding stability (Stand); and (7) performance errors like misfitting parts, incomplete connections, or dropping pieces without attempting to recover them (Errors). The complex score (Comp) is the sum of these variables.

**Table 2:** Scoring criteria.

Variable	Scoring criteria	Points
1. MC	Made constructions: Created using any combination of pieces ＝ 1	1
2. NP	Number of pieces used (*n*) 0–19 ＝ 1, 20–39 ＝ 2, 40–49 ＝ 3, Exactly 50 ＝ 4 Note: “*n*” denotes the total number of pieces used in the creation process.	1–4
3. Name	Appropriate ＝ 3; Vague/inappropriate ＝ 2; post‐hoc naming, description ＝ 1，none ＝ 0	0–3
4. MOV	Mobility ＝ 1，moving parts ＝ 1	0–2
5. 3D	Three‐dimensional	1
6. Stand	Free‐standing, stays standing	1
7. Errors	For each error (misfit, incomplete fit, dropped and not picked up)	−1
8. Scoring for creation process	The participant did not have a creation image envisaged and completed or the participant did not know what was created with Tinkertoy ＝ 0. The participant was unable to create with a clear image and completed a portion of some kind of work ＝ 1. The participant created images in the process of assembly, created work and finished it ＝ 2. The participant created images, created work and finished it ＝ 3. The participant created and completed the work based on the surrounding environment ＝ 4.	0–4
Highest score possible		16
Lowest score possible		−1 or less

The creation process score was calculated on the following scale: a score of 0 was given when the participant had no creative vision and completed the assembly without a clear understanding of what they had constructed. A score of 1 was assigned when the participant lacked a clear creative image but managed to complete some form of the work. A score of 2 indicated that the participant developed a creative image during the assembly process and finished the construction accordingly. A score of 3 was given when the participant had a clear creative image from the outset and followed up to complete the work. Finally, a score of 4 was awarded when the participant not only had a clear creative image but also considered the surrounding environment in their construction. To obtain the total score for the m‐TTT, we summed the scores for both the complexity and creation process.

##### Other DT tests

Two other DT assessments (IFT and DFT) were administered. In the IFT, the examiner asked participants to think of as many uses as possible for an empty tin within 5 min. In this test, we classified the responses into the following three categories. The first was task‐dependent (Idea Td): ideas based on the tin's nature as a container (e.g., a pen stand). The second was task‐modified (Idea Tm): ideas free from the tin's nature as a container but still retaining an original form (e.g., “kick‐the‐can”). The third was task‐independent (Idea Ti): all ideas except for the task‐dependent and task‐modified ones (e.g., accessories).^24^

In the DFT, the examiner asked participants to produce as many different drawings as possible for one set of four dots within 3 min. Upon completion of the task, the examiner classified the drawings into the following three categories. The first was task‐dependent (Design *T*_d_): drawings based on a geometrical pattern. The second was task‐modified (Design *T*_m_): drawings based on the shape of a square. The third was task‐independent (Design *T*_i_): the rest of the drawings, except for the task‐dependent and task‐modified ones.^25^

#### Neurocognitive measures

Neuropsychological performance, measured by the Japanese version of the Brief Assessment of Cognition in Schizophrenia (BACS)^26^ was evaluated by experienced psychiatrists or psychologists. The BACS measures various components, including list learning (verbal memory), digit sequencing tasks (working memory), token motor tasks (motor function), category and letter fluency (verbal fluency), symbol coding (attention and processing speed), and the Tower of London test (executive function). The composite score is calculated based on the average *z*‐score for each item.

#### Assessment of life skills

To assess life skills, we used the Life Skills Profile (LSP) developed by Rosen et al.^27^ to measure disabilities in daily life and community functioning in patients with chronic mental disorders. The reliability and validity of this scale have been previously confirmed.^27^, ^28^ Family members, psychiatric professionals, and case workers can be used as informants in the interview.^29^ This instrument consists of five subscales comprising 39 items. Each item is rated on a scale from 1 to 4. The five subscales are Self‐Care, Nonturbulence, Socialization, Communication, and Responsibility. Higher scores indicate better functional outcomes. Given the characteristics of these scales, they were only administered to the schizophrenia group.

#### Psychiatric symptoms

The Positive and Negative Syndrome Scale (PANSS)^30^ was used to obtain scores on the Positive Symptoms subscale, Negative Symptoms subscale, and General Psychopathology subscale. Given the characteristics of these scales, they were only administered to the schizophrenia group.

### Statistical analysis

All statistical analyses were conducted using SPSS Version 21.0 for Windows. Initially, *t*‐tests and Mann–Whitney *U*‐tests were employed to investigate the differences in the m‐TTT complex score, creation process score, total score, and other DT tests and neurocognitive measures between the patient and control groups. Subsequently, to elucidate the significant associations between the LSP total scores and other clinical variables, Pearson's correlation coefficients were calculated. Stepwise regression analyses were then conducted for clinical variables that showed significant correlations with the LSP total score to identify the best predictors. Only variables meeting our inclusion threshold of a *p*‐value of <0.05 for initial correlations with the LSP total score were considered for these analyses. This rigorous selection ensures that only statistically significant predictors are included in the model.

## RESULTS

The mean scores on the PANSS subscales in the patient group were as follows: Positive Symptoms, 18.6 (SD = 4.9); Negative Symptoms, 18.1 (SD = 4.4); and General Psychopathology, 37.2 (SD = 6.6). The mean scores for the life skills measures were: LSP total score, 127.1 (SD = 11.0); LSP self‐care score 29.4 (SD = 4.2); LSP nonturbulence score, 44.9 (SD = 3.3); LSP social contact score, 14.9 (SD = 3.3); LSP communication score, 20.0 (SD = 2.2); and LSP responsibility score, 17.8 (SD = 2.1).

### Differences in neurocognitive measures between the schizophrenia and control groups

The results of the between‐group analysis are shown in Table 3. Significant differences were observed across various metrics. Specifically, the patient group scored significantly lower in terms of the m‐TTT complexity, creation process, and overall (*p* < 0.001).

**Table 3:** Comparison of m‐TTT, other divergent thinking tests, and BACS between the schizophrenia and control groups.

	Schizophrenia group	Neurotypical control group
	(*n* ＝ 52)	(*n* ＝ 51)
*m‐TTT*		
Time required for testing (s)	387 (300–4029)	402 (300–1310)
Complex	7.0 (2.3)***	8.6 (2.1)
Creation process	1.3 (1.1)***	2.3 (2.0)
Total	8.3 (3.2)***	10.9 (2.9)
*IFT*		
Task‐dependent	4.7 (2.7)***	7.5 (4.6)
Task‐modified	2.5 (1.8)***	4.5 (2.9)
Task‐independent	2.2 (2.6)	2.6 (2.3)
*DFT*		
Task‐dependent	10.9 (7.8)*	15.7 (10.4)
Task‐modified	3.3 (4.8)	5.1 (6.0)
Task‐independent	3.4 (4.2)	3.8 (6.2)
*BACS*		
Composite score	−1.5 (1.4)***	0.2 (0.9)

*Note*: For m‐TTT, the time required for testing (s) is presented as the median (with minimum to maximum values in parentheses). For all other metrics, values are presented as means (with standard deviations in parentheses).

Abbreviations: BACS, Brief Assessment of Cognition in Schizophrenia; DFT, Design Fluency Test; IFT, Idea Fluency Test; m‐TTT, modified Tinkertoy Test.

*** *p* ＜ 0.001;

* *p* < 0.05.

In the IFT, no significant differences were found in the task‐independent scores between the two groups; however, the patient group scored lower in the task‐dependent and task‐modified scores (*p* < 0.001) compared with the control group.

Similarly, in the DFT, the patient group had a significantly lower task‐dependent response score (*p* < 0.05). Furthermore, the BACS revealed that the patient group had significant reductions in composite scores (*p* < 0.001) compared with the control group.

### Correlations between community functioning and neurocognitive/demographic/clinical variables

The LSP total score significantly correlated with the total m‐TTT (*r* = 0.417, *p* < 0.01), complexity (*r* = 0.398, *p* < 0.01), and creation process scores (*r* = 0.413, *p* < 0.01); BACS composite Z‐score (*r* = 0.278, *p* < 0.05); PANSS Negative Symptoms score (*r* = −0.437, *p* < 0.001), and PANSS General Psychopathology score (*r* = −0.399, *p* < 0.01). To demonstrate the independence of the scales that showed significant correlations with the LSP total score, we found no significant correlations among these scales (Table 4).

**Table 4:** Correlations between Life Skills Profile (LSP) total score and measures of neurocognitive performance and psychiatric symptoms.

	LSP total score
*m‐TTT*	
Complex score	0.398**
Creation process	0.413**
Total score	0.417**
*IFT*	
Task‐dependent	0.142
Task‐modified	0.215
Task‐independent	0.236
*DFT*	
Task‐dependent	−0.198
Task‐modified	0.139
Task‐independent	0.212
*BACS*	
Composite score	0.278*
*PANSS*	
Positive Symptoms	−0.119
Negative Symptoms	−0.437***
General Psychopathology	−0.399**

*Note*: Score correlations as shown were bivariate Pearson correlation coefficients.

Abbreviations: BACS, Brief Assessment of Cognition in Schizophrenia; DFT, Design Fluency Test; IFT, Idea Fluency Test; LSP, Life Skills Profile; m‐TTT, modified Tinkertoy Test; PANSS, Positive and Negative Syndrome Scale.

*** *p* < 0.001;

** *p* < 0.01;

* *p* < 0.05.

No significant correlations were found between the LSP total score and age, education, duration of disease, Japanese Adult Reading Test (JART), antipsychotics daily dosage level (mg/day), PANSS Positive Symptoms, IFT task‐dependent, task‐modified, task‐independent, DFT task‐dependent, task‐modified, or task‐independent variables in either the IFT or DFT.

Furthermore, no correlation was identified between age and the m‐TTT total score in either the schizophrenia or neurotypical control groups. Additionally, no gender differences in the m‐TTT total score were observed in either group.

### Correlation between the LSP total score and items that show correlation and correlation between m‐TTT and other DT measures

Correlations were identified between the LSP total score and other metrics as follows: the m‐TTT total score and the complex score (*r* = 0.985, *p* < 0.001) and the m‐TTT total score and the creation process (*r* = 0.929, *p* < 0.001). Significant correlations were also noted between PANSS Negative Symptoms and General Psychopathology (*r* = 0.540, *p* < 0.001). No significant correlations were detected among the other items. Significant correlations were noted between the m‐TTT total score and both the IFT task‐modified (*r* = −0.376, *p* < 0.01) and task‐independent (*r* = −0.470, *p* < 0.01). No significant correlations were detected with other DT measures.

### Contribution of DT deficits to life skills

We conducted a stepwise regression analysis using predictors that demonstrated significant correlations with the total LSP score, specifically the m‐TTT total score, complexity score, creation process, BACS composite score, PANSS Negative Symptoms, and PANSS General Psychopathology. The model for the total LSP score was significant (*R*² = 0.309, *p* < 0.01), including two primary variables: the m‐TTT total score (Beta=0.349, *p* = 0.006) and the PANSS Negative Symptoms score (Beta = −0.374, *p* = 0.003). The final variables identified by the stepwise multiple regression analysis as influencing the LSP total score were the m‐TTT total score and the PANSS Negative Symptoms score. This indicates that a higher m‐TTT total score is associated with better life skills, and a lower PANSS Negative Symptoms score correlates with improved life skills. In the revised analysis, we further scrutinized the relationships among the variables using additional statistical tests to understand the lack of inclusion of some variables with significant univariate correlations. For instance, despite its significant correlation with LSP, General Psychopathology was not included in the final model owing to its collinearity with PANSS Negative Symptoms, which was evidenced by a high variance inflation factor. Such statistical nuances are critical in stepwise regression to avoid overfitting and to ensure the model's generalizability.

## DISCUSSION

In this study, we compared the cognitive functions of patients with schizophrenia to those of a neurotypical control group. The results revealed that the former scored significantly lower on the m‐TTT, IFT, and DFT, all of which measured DT. Furthermore, their BACS composite scores, which are indicative of overall cognitive functions, were also lower. The most significant factors affecting the total LSP scores in these patients were the total m‐TTT score and severity of negative symptoms.

For the DT tasks, it was evident that the scores of the schizophrenia group were lower in the m‐TTT creation process, complex total, IFT task‐dependent and task‐modified, and the DFT task‐dependent scores. Prior research has highlighted a negative correlation between schizophrenia and creativity, including DT. It has been stated that the performance of the verbal scale of DT is lower than that of the nonverbal scale.^10^ In this study, many significant differences were observed on the verbal scale of the IFT. Furthermore, given the significant differences observed in the m‐TTT, which requires both verbal and nonverbal skills, this suggests that a measure that integrates both linguistic and nonlinguistic factors is effective in assessing DT. Previous studies on DT have shown that it is characterized by selective and flexible inhibition and changes according to the focus of attention and demands of the task.^31^ DT decreases inhibition in the initial stages of a task and selects the relevant information from a vast array of unrelated information. In the later stages, it enhances inhibition while narrowing the focus of attention to tackle the task.^32^, ^33^

The m‐TTT used in this study required the participants to autonomously engage in continuous task execution from start to finish. This open‐ended task demands the ability to switch between inhibition and noninhibition, reflecting a key characteristic of DT. Moreover, literature suggests that DT assessments conducted without time constraints tend to show enhanced performance compared to those with time limitations.^34^ The lack of a time restriction in the m‐TTT underscores its potential utility in evaluating DT. The m‐TTT, an untimed DT test, showed no significant differences in the time required for testing between the schizophrenia group and the neurotypical control group. Additionally, it was found that in both groups, the majority of assessments were completed in less than 10 min. These results suggest that, although m‐TTT does not impose a time limit, it is a practical test that can generally be administered within approximately 10 min. However, there was considerable variability in test durations within the schizophrenia group, with some participants requiring extended periods to complete the test. This variability indicates that modifications might be necessary for participants who are unable to complete the task within the typical timeframe.

Previous studies have reported a correlation between the community living skills of patients with schizophrenia and scores on the IFT's task‐independent and task‐modified, as well as the DFT task‐modified scores.^6^ This study revealed that the m‐TTT total score is a factor influencing the LSP total score. Furthermore, within the schizophrenia group, correlations were observed between the m‐TTT and scores on the IFT's task‐independent and task‐modified. These findings suggest that the m‐TTT, adopted as a DT measure in this study, may offer higher accuracy in predicting life skills compared to other DT measures. This could be attributed to the m‐TTT's free composition task requiring both nonverbal and verbal DT elements, thereby identifying it as a significant factor beyond traditional DT measures.

The results of this study suggest that both the total m‐TTT score and PANSS Negative Symptoms score are predictive of the total LSP score in patients with schizophrenia. Notably, the total m‐TTT score emerged as a more potent predictor of life skills than the TTT complex and IFT scores. Both these scores have been identified in previous studies as being linked to the daily life adaptation of patients with schizophrenia.^6^, ^21^ Moreover, no correlation was observed between the m‐TTT total score and PANSS Negative Symptoms score, indicating that each variable independently affects the LSP total score.

The total m‐TTT score is derived by adding the traditional complex score, which assesses finished work against established criteria, and the newly introduced creation process score. The latter evaluated the spontaneous initiation, planning, execution, and monitoring of tasks in a free‐form context, complementing the complex score and thereby enhancing the accuracy of DT measurements in patients with schizophrenia.^20^ Furthermore, the results of this study revealed that corroboration of both evaluation items is necessary to predict life skills. This highlights the value of the m‐TTT total score, which can measure both functions collaboratively.

Our findings suggest that negative symptoms of schizophrenia, particularly diminished motivation, may adversely affect engagement in life skills. This aligns with previous research indicating that such symptoms negatively impact community living.^34^, ^35^, ^36^, ^37^, ^38^, ^39^ Moreover, other studies have demonstrated that intrinsic motivation significantly influences social functioning, independent of negative symptoms.^40^ However, our study did not measure intrinsic motivation directly, and elucidating the relationship between intrinsic motivation and divergent thinking represents an important direction for future research.

This study underscores the exploratory evaluation of DT, as assessed by the m‐TTT, in evaluating life skills among patients with schizophrenia, relative to other cognitive function assessments. It offers initial insights into the complex nature of life skills assessment within this population. First, it acknowledges that while traditional life skills tests frequently utilize role‐play to simulate real‐world scenarios, these methods encounter challenges, including biases potentially introduced by age and cultural differences.^41^ The m‐TTT, through its open‐ended tasks, proposes an alternative approach that could yield insights into life skills through the lens of DT, mitigating biases related to these factors. Second, the findings suggest the potential advantages of DT‐centered interventions in enhancing life skills for individuals with schizophrenia. Nonetheless, these findings should be interpreted with caution given the study's cross‐sectional design and the varied correlations observed between different DT measures and life skills performance. Further longitudinal research is needed to validate these tentative relationships and assess the effectiveness of DT‐focused interventions in this context.

This study had several limitations. First, owing to its cross‐sectional design, it was not possible to establish a causal relationship between clinical variables and life skills. Second, the sample size was small. Further research with a larger sample size is necessary to validate our findings. Third, a significant limitation of this study is the absence of validated inter‐rater reliability for the scoring system used in the m‐TTT. This is critical as it questions the consistency and reproducibility of the test results, emphasizing the need for future studies to establish and report the reliability of these measures. In addition to establishing inter‐rater reliability, future research should also focus on validating the m‐TTT across diverse clinical and nonclinical populations to ascertain its generalizability and utility in different settings. Fourth, the sample consisted entirely of stable outpatients, which may limit the generalizability of the results to a broader population of individuals with schizophrenia.

## CONCLUSIONS

In our study, the m‐TTT and other DT tests demonstrated a diminished capacity for DT in individuals with schizophrenia compared to the neurotypical controls. Additionally, the m‐TTT scores and negative symptoms of schizophrenia were identified as significant factors affecting the overall LSP score. These findings underscore the pivotal role of DT in improving life skills in patients with schizophrenia. Interventions targeting these cognitive functions can enhance community adaptation skills.

## AUTHOR CONTRIBUTIONS

Yasuhisa Nakamura conceptualized and designed the study, performed the analysis, and drafted the manuscript. Yasuhisa Nakamura is also the corresponding author responsible for communication with the journal during the manuscript submission, peer review, and publication process. Reiko Miyamoto and Akihiro Koreki collected the data, assisted with data analysis, and contributed to the writing and editing of the manuscript. Sachiko Anamizu and Masaru Mimura contributed to the study design, provided critical revisions of the manuscript, and supervised the project.

## CONFLICT OF INTEREST STATEMENT

The last author (Masaru Mimura) is an Editorial Board member of *Psychiatry and Clinical Neurosciences Reports* and a co‐author of this article. To minimize bias, they were excluded from all editorial decision‐making related to the acceptance of this article for publication. Masaru Mimura declares no conflict of interest directly related to this work. The first author (Yasuhisa Nakamura) declares no conflict of interest.

## ETHICS APPROVAL STATEMENT

The study protocol was approved by the Clinical Research Ethics Committee of Nihon Fukushi University, Japan (15‐30). The trial was registered with the UMIN Clinical Trials Registry (UMIN000031879).

## PATIENT CONSENT STATEMENT

After providing them with a complete description of the study, written informed consent was obtained from each participant.

## CLINICAL TRIAL REGISTRATION

The trial was registered with the UMIN Clinical Trials Registry (UMIN000031879).

## References

[1] Bellack AS , Morrison RL , Wixted JT , Mueser KT . An analysis of social competence in schizophrenia. Br J Psychiatry. 1990;156:809–818. 2207511 10.1192/bjp.156.6.809

[2] Bozikas VP , Kosmidis MH , Kafantari A , Gamvrula K , Vasiliadou E , Petrikis P , et al. Community dysfunction in schizophrenia: rate‐limiting factors. Prog Neuropsychopharmacol Biol Psychiatry. 2006;30:463–470. 16442195 10.1016/j.pnpbp.2005.11.017

[3] Puig O , Penadés R , Baeza I , De la Serna E , Sánchez‐Gistau V , Lázaro L , et al. Assessment of real‐world daily‐living skills in early‐onset schizophrenia trough the Life Skills Profile scale. Schizophr Res. 2013;145(1–3):95–100. 23384737 10.1016/j.schres.2012.12.026

[4] Tyson PJ , Laws KR , Flowers KA , Mortimer AM , Schulz J . Attention and executive function in people with schizophrenia: relationship with social skills and quality of life. Int J Psychiatry Clin Pract. 2008;12(2):112–119. 24916621 10.1080/13651500701687133

[5] Tominaga T , Tomotake M , Takeda T , Ueoka Y , Tanaka T , Watanabe S , et al. Predictors of life skills in people with schizophrenia. J Med Invest. 2020;67(1):75–82 32378622 10.2152/jmi.67.75

[6] Nemoto T , Kashima H , Mizuno M . Contribution of divergent thinking to community functioning in schizophrenia. Prog Neuropsychopharmacol Biol Psychiatry. 2007;31(2):517–524. 17218048 10.1016/j.pnpbp.2006.12.001

[7] Goldschmidt G . Linkographic evidence for concurrent divergent and convergent Thinking in creative design. Creat Res J. 2016;28(2):115–122.

[8] Webb M , Little DR , Cropper S , Roze K . The contributions of convergent thinking, divergent thinking, and schizotypy to solving insight and non‐insight problems. Think Reason. 2007;23(3):1–24.

[9] Palmiero M , Fusi G , Crepaldi M , Borsa VM , Rusconi ML . Divergent thinking and the core executive functions: a state‐of‐the‐art review. Cogn Process. 2022;23(3):341–366. 35461411 10.1007/s10339-022-01091-4

[10] Acar S , Chen X , Cayirdag N . Schizophrenia and creativity: a meta‐analytic review. Schizophr Res. 2018;195:23–31. 28867517 10.1016/j.schres.2017.08.036

[11] Lezak MD . The problem of assessing executive functions. Int J Psychol. 1982;17:281–297.

[12] Lezak MD . Neuropsychological assessment. New York: Oxford University Press; 2012. p. 684–689.

[13] Crippa F , Cesana L , Daini R . Normative data for Lezak's Tinkertoy test in healthy Italian adults. F1000Res. 2016;5:727.

[14] Bayless JD , Varney NR , Roberts RJ . Tinkertoy test performance and vocational outcome in patients with closed‐head injuries. J Clin Exp Neuropsychol. 1989;11:913–917. 2592530 10.1080/01688638908400944

[15] Cicerone KD , De Lucca J . Neuropsychological predictors of head injury rehabilitation outcome. J Clin Exp Neuropsychol. 1990;12:92.

[16] Roberts MA , Franzen K , Furuseth A , Fuller L . A developmental study of the Tinker Toy® test: normative and clinical observations. Appl Neuropsychol. 1995;2:161–166. 16318521 10.1080/09084282.1995.9645355

[17] Mendez MF , Ashla‐Mendez M . Differences between multi‐infarct dementia and Alzheimer's disease on unstructured neuropsychological tasks. J Clin Exp Neuropsychol. 1991;13:923–932. 1779031 10.1080/01688639108405108

[18] Koss E , Patterson MB , Mack JL , Smyth KA , Whitehouse PJ . Reliability and validity of the Tinkertoy test in evaluating individuals with Alzheimer's disease. Clin Neuropsychol. 1998;12:325–329.

[19] Ownsworth T , Shum D . Relationship between executive functions and productivity outcomes following stroke. Disabil Rehabil. 2008;30:531–540. 17852299 10.1080/09638280701355694

[20] Nakamura Y , Yamanaka T , Ishii F , Anamizu S , Mimura M . Reliability and validity of modified the Tinkertoy test in schizophrenia patients. High Brain Funct Res. 2019;39(1):10–17.

[21] Christensen K , Mateer C , Williams R , Woodward T . Neuropsychological deficits, syndromes, and cognitive competency in schizophrenia. Cognit Neuropsychiatry. 2005;10:361–378. 16571467 10.1080/13546800444000100

[22] American Psychiatric Association . (2013). Diagnostic and statistical manual of mental disorders (5th ed.). Washington, DC: American Psychiatric Association.

[23] Oldfield RC . The assessment and analysis of handedness: the Edinburgh Inventory. Neuropsychologia. 1971;9:97–113. 5146491 10.1016/0028-3932(71)90067-4

[24] Nemoto T , Mizuno M , Kashima H . Qualitative evaluation of divergent thinking in patients with schizophrenia. Behav Neurol. 2005;16(4):217–224. 16518012 10.1155/2005/386932 PMC5478849

[25] Keefe R . The brief assessment of cognition in schizophrenia: reliability, sensitivity, and comparison with a standard neurocognitive battery. Schizophr Res. 2004;68:283–297. 15099610 10.1016/j.schres.2003.09.011

[26] Kaneda Y , Sumiyoshi T , Keefe R , Ishimoto Y , Numata S , Ohmori T . Brief assessment of cognition in schizophrenia: validation of the Japanese version. Psychiatry Clin Neurosci. 2007;61(6):602–609. 18081619 10.1111/j.1440-1819.2007.01725.x

[27] Rosen A , Hadzi‐Pavlovic D , Parker G . The Life Skills Profile: a measure assessing function and disability in schizophrenia. Schizophr Bull. 1989;15:325–337. 2749191 10.1093/schbul/15.2.325

[28] Hasegawa K , Ogawa K , Kondoh C , Iseda T , Ikebuchi T , Miyake Y . The reliability and validity of the Japanese version of the Life Skills Profile (in Japanese). Seishin Igaku. 1997;39:547–555.

[29] Leifker FR , Patterson TL , Heaton RK , Harvey PD . Validating measures of real‐world outcome: the results of the VALERO expert survey and RAND panel. Schizophr Bull. 2011;37:334–343. 19525354 10.1093/schbul/sbp044 PMC3044614

[30] Kay SR , Fiszbein A , Opler LA . The positive and negative syndrome scale (PANSS) for schizophrenia. Schizophr Bull. 1987;13:261–276. 3616518 10.1093/schbul/13.2.261

[31] Vartanian O , Martindale C , Matthews J . Divergent thinking ability is related to faster relatedness judgments. Psychol Aesthet Creat Arts. 2009;3:99–103.

[32] Beaty RE , Silvia PJ . Why do ideas get more creative across time? An executive interpretation of the serial order effect in divergent thinking tasks. Psychol Aesthet Creat Arts. 2012;6(4):309–319.

[33] Zabelina DL , Ganis G . Creativity and cognitive control: behavioral and ERP evidence that divergent thinking, but not real‐life creative achievement, relates to better cognitive control. Neuropsychologia. 2018;118(Part A):20–28. 29447843 10.1016/j.neuropsychologia.2018.02.014

[34] Paek SH , Abdulla Alabbasi AM , Acar S , Runco MA . Is more time better for divergent thinking? A meta‐analysis of the time‐on‐task effect on divergent thinking. Think Skill Creat. 2021;41:100894.

[35] Aubin G , Stip E , Gélinas I , Rainville C , Chapparo C . Daily activities, cognition and community functioning in persons with schizophrenia. Schizophr Res. 2009;107(2–3):313–318. 18824328 10.1016/j.schres.2008.08.002

[36] Snyder PJ , Jackson CE , Piskulic D , Olver J , Norman T , Maruff P . Spatial working memory and problem solving in schizophrenia: the effect of symptom stabilization with atypical antipsychotic medication. Psychiatry Res. 2008;160(3):316–326. 18579217 10.1016/j.psychres.2007.07.011

[37] Huang J , Tan S , Walsh SC , Spriggens LK , Neumann DL , Shum DHK , et al. Working memory dysfunctions predict social problem solving skills in schizophrenia. Psychiatry Res. 2014;220(1–2):96–101. 25110314 10.1016/j.psychres.2014.07.043

[38] Kaizerman‐Dinerman A , Roe D , Demeter N , Josman N . Do symptoms moderate the association between participation and executive functions outcomes among people with schizophrenia? BMC Psychiatry. 2023;23(1):42. 36650458 10.1186/s12888-022-04510-0 PMC9844002

[39] Becker TM , Cicero DC , Cowan N , Kerns JG . Cognitive control components and speech symptoms in people with schizophrenia. Psychiatry Res. 2012;196(1):20–26. 22365272 10.1016/j.psychres.2011.10.003 PMC4445960

[40] Yamada AM , Lee KK , Dinh TQ , Barrio C , Brekke JS . Intrinsic motivation as a mediator of relationships between symptoms and functioning among individuals with schizophrenia spectrum disorders in a diverse urban community. J Nerv Ment Dis. 2010;198(1):28–34. 20061866 10.1097/NMD.0b013e3181c8aa71 PMC2946838

[41] Mausbach BT , Tiznado D , Cardenas V , Jeste D , Patterson T Validation of the UCSD Performance‐based Skills Assessment (UPSA) in Hispanics with and without schizophrenia. Psychiatry Res. 2016; 30(244):388–393. 10.1016/j.psychres.2016.08.027 PMC5026959 27525829

